# Comments of “W. H.,” Assistant-Editor of the Chicago Medical Journal, Feb., 1871, upon Opium in Metro-Peritonitis, Briefly Reviewed

**Published:** 1871-03

**Authors:** Theodore Griffin

**Affiliations:** Chicago, Illinois


### COMMENTS OF "W. H.," ASSISTANT-EDITOR OF THE CHICAGO MEDICAL JOURNAL, FEB., 1871, UPON OPIUM IN METRO-PERITONITIS, BRIEFLY REVIEWED.

By THEODORE GRIFFIN, M.D., Chicago, Illinois.

The article on "Opium in Metro-Peritonitis," which I contributed to THE EXAMINER for December, 1870, is quoted in full in the *Chicago Medical Journal* for February, 1871, under "Selections" (Walter Hay, assistant-editor) followed, by what was no doubt designed to be a scathing criticism thereof. The article appears over the initial letters, "W. H.," and is markedly characterized by a labored effort, as I have said, to be *scathing*.

The writer begins his criticism and argument (?) by an assault upon the editor of THE EXAMINER for admitting my report for publication; stating that such articles are calculated to mislead the unwary, and possibly to occasion disastrous results. He accuses the worthy editor of THE EXAMINER of "conducting a reckless style of journalism." Then follows some rambling generalizing about opium eating, after which the writer directs his remarks to the report in question. The second paragraph of this article is devoted to a condemnation of the manner in which I *observe* my cases. In the third paragraph the writer attempts to convince his readers, by an improper arrangement of sentences and portions of sentences, belonging to different paragraphs of my report, that I regarded "bowels constipated," pulse 90 and bounding," etc., as a plain indication for the use of opium. "Which" (symptoms) says this erudite writer, "according to the teachings of modern physiology contraindicate its use." While the symptoms above-named were present, no opium was given; these precursory symptoms yielded, as I said, to a little mild treatment without the employment of opium. In the fourth paragraph the writer defines an axiom, and states, that the axiom which I proposed "involves both a verbal and therapeutic paradox, the second and third terms each containing a possible contradiction of the first." How this can be is beyond my comprehension, as the axiom contains but *two* terms, and not *three*, as is asserted by the *learned* critic. I, however, shall quote the *axiom* which so much agitated the mind of "W. H.," at the close of this article, that the reader may exercise his mind in a search for the "three terms" thereof, as well as for the "verbal and therapeutic paradox," said to be contained therein. Now follows, in the two succeeding paragraphs, a somewhat detailed notice of the, to him, incomprehensible use of the large and increasing doses of opium which I employed. Observe what he says: "Although the patient was much worse, and her '*nervous system much disturbed and agitated*,' (italics his), the dose was rather more than doubled." I increased the dose from one to two grains per hour, as "the pain continued unabated, and her appearance indicated great suffering." "W. H.," however, is startled at the idea of increasing the dose under such circumstances. "W. H." becomes more and more horrified as the size of the dose is continuously increased to vi grs. of opium per hour—which was the maximum dose employed—and he finally exclaims: "Was she better? more opium! Was she unchanged? more opium!! Was she worse? still more opium!!!"

The writer says the analysis of the case suggests the following subjects of inquiry, the second subject being, as he tells us, "the total failure of the opium to produce any of its constitutional reactions, aside from 'disturbance and agitation of the nervous system,' 'subsultus tendinum,' and arrest of the renal and 'mammary secretions,' which the author evidently excludes from that category." Thus the reader will perceive that the writer mistakes the symytoms produced by the disease for the "constitutional reactions" of the opium.

The 3d subject of inquiry is into "the persistence in the administration of a drug which had so entirely failed, not only to meet the indications, but even to manifest any of its usual effects."

This 3d subject of inquiry will be considered in a brief notice of the Use of Opium in Metro-Peritonitis, at the close of this article.

The writer declares in closing, that either,

1st, *The medicine was not taken*, or,

2d, That the drug was worthless, or,

3d, That the medicine was passed unchanged in the stools, or,

4th, Which he believes to be the true solution of the mystery, the woman was an opium eater already.

Throughout the whole of the article "W. H." exhibits a vindictive style of expression which is truly impressive; he also displays a remarkable degree of ignorance of that portion of medical literature which relates to metro-peritonitis and its treatment. For instance, he mentions the nervous agitation, subsultus tendinum, diminished renal and mammary secretions, as "constitutional reactions" of the opium which was given; whereas, every intelligent medical student knows that these are prominent and ever present symptoms in severe cases of metro-peritonitis.

I affirm, 1st, That opium was taken in the case represented by me, in the quantity, and in the time mentioned.

2d, That the opium taken was *good* opium.

3d, That to the best of my knowledge the woman was not an opium eater, and

4th, That the patient made a rapid and satisfactory recovery, which is the end most desired in treatment.

For the satisfaction of "W. H.," who flatly asserts that I was guilty of misrepresentation, or that the woman was an opium eater, I will brieflly allude to the experience of Prof. Alonzo Clark in the treatment of this disease with opium, as detailed in Prof. Byford's Medical and Surgical Treatment of Women, page 536, to show that a patient laboring under puerperal peritonitis, may take not only vi grs. of opium per hour without harm, but may take more than three times that amount, for many successive hours, with benefit.

Alonzo Clark says, "Puerperal peritonitis has frequently visited the lying-in wards of Bellevue Hospital during the last twenty years, and the recovery of those who have been attacked by it, up to the winter of 1851–2, was the exception rather than the rule. Having acquired great confidence in the efficacy of large doses of opium in simple peritonitis, when the puerperal form of the disease made its appearance in December, 1857, I resolved to try its virtues in this more formidable affection." Then follows a detailed report of a number of cases. It will answer my present purpose to mention the amount of of opium given in two out of a number of cases, illustrating, as they do, the great tolerance of opium in this disease. In one case "xij grs. of opium were given per hour, for many successive hours, without marked narcotism." In the seventh case reported, "the treatment was commenced at 10 A.M., on the 26th of December. Two grs. of opium were given hourly; at 2 P.M. no change in symptoms, dose increased to iv grs. per hour; at 4 P.M. to v grs. hourly; at 6 P.M. to viij grs.; at 8 P.M. to x grs.; at 9 P.M. to xij grs.; and so on to the termination of the disease." "This woman took in the second 24 hours of her treatment 472 grs. of opium," averaging $19\frac{7}{12}$ grs. of opium per hour. "On the third day she took 236 grs. of opium." "She assured me repeatedly," says Dr. Clark, "that she did not know opium by sight, and had never taken it or any of its preparations unless prescribed by a physician."

The two patients above alluded to recovered.

As a result of his experience with opium in puerperal peritonitis, Dr. Clark gives us the following conclusions:

"1st. When a prominent element in puerperal fever is peritonitis, the treatment with large doses of opium is more successful than any other which has been proposed."

"2d. To be successful, this treatment must be commenced early, and the patient must be brought under its influence as rapidly as the susceptibility of the system can be ascertained by trial."

"3d. The quantity of opium required to produce a safe but desirable degree of narcotism varies greatly in different cases, so that it is necessary to begin with doses that cannot do mischief, and increase every two hours until the influence of the opiate is sufficiently decided."

"6th. The tolerance of opium in some cases of puerperal peritonitis almost surpasses belief," etc.

"7th. The influence of the opium should be kept up until the pain and tenderness subsides."

In the treatment of the case reported by me in the December number of THE EXAMINER for 1870, I was guided by essentially the same ideas as are expressed in Dr. Clark's conclusions; and were I called to treat a case of metro-peritonitis to-day, I should use opium; and I should use it as directed in the 2d and 7th paragraphs of Dr. Clark's conclusions.

Why this great tolerance of opium exists in this disease is not readily accounted for; it may doubtless be accounted for in part upon the hypothesis that pain, which is present in an intense degree when there is a great amount of peritoneal inflammation, may counteract the physiological influence of the drug upon the nervous system. Pain, the reader is aware, is due to some disturbance of the nervous system. Opium, the reader is also aware, exerts its effect primarily upon the nervous system, and secondarily, through the nervous system upon the circulation, organs of respiration, etc.

It therefore follows, that while the nervous system is laboring under an intense degree of irritation, denominated *pain*, that, great quantities of opium taken into the stomach and absorbed, would most probably be inadequate to produce the poisonous effect of opium; whereas, when the nervous system is in a natural and placid state, how readily it yields, and is overwhelmed by the sedative effect of the opium.

It is needless to warn the intelligent physician to be careful in the use of so potent an agent as opium; he is already apprised of its power, and knows how far it can be administered with safety; "W. H.'s" assertion to the contrary notwithstanding.

Let this fact, which should be accepted as an *axiom*, be constantly borne in mind at the bedside: "*If indications are scrupulously met in treatment, no drug poisoning, with its disagreeable and dangerous perturbations, will occur, however large the dose of the drug may be.*"

I wish, in conclusion, to call attention to one other paragraph by "W. H.," when, referring to my use of opium, he questions "(1st) the propriety of administering a cardiac stimulant and arterial sedative (?) when the reciprocating equilibrium between these two portions of the circulatory system was already disturbed by the increased activity of the heart."

Is it true, that opium is *at the same time* a "*cardiac stimulant and arterial sedative*"? Here is a "therapeutic paradox" in reality.

I trust that I have made apparent, what every intelligent physician must know who has read this critic's criticism; that the critic is deplorably ignorant of the subject upon which he presumes to criticise.

